# Clinical outcomes associated with antidepressant use in inflammatory bowel disease patients and a matched control cohort

**DOI:** 10.1038/s41598-024-51282-6

**Published:** 2024-01-11

**Authors:** Djibril M. Ba, Sanjay Yadav, Guodong Liu, Douglas L. Leslie, Kent E. Vrana, Matthew D. Coates

**Affiliations:** 1 Department of Public Health Sciences, Pennsylvania State University College of Medicine, Hershey, PA USA; 2 Center for Applied Studies in Health Economics (CASHE), Pennsylvania State University College of Medicine, Hershey, PA USA; 3 Department of Psychiatry, Pennsylvania State University College of Medicine, Hershey, PA USA; 4 Department of Pharmacology, Pennsylvania State University College of Medicine, Hershey, PA USA; 5 Department of Medicine, Division of Gastroenterology and Hepatology, Pennsylvania State University College of Medicine, Hershey, PA USA; 6 Penn State-Hershey Medical Center, 500 University Dr., Hershey, PA 17033 USA

**Keywords:** Depression, Inflammatory bowel disease

## Abstract

Antidepressant medications (AMs) are frequently used in inflammatory bowel disease (IBD). Many AMs enhance serotonin (5-HT) availability, but this phenomenon may actually worsen IBD. We hypothesized that use of 5-HT-enhancing AMs would be associated with poor clinical outcomes in these disorders. We performed a retrospective cohort study using the Merative Health Marketscan® commercial claims database between 1/1/05 and 12/31/14. Participants (18–63 years) were either controls or had ≥ 2 ICD-9 diagnoses for IBD with ≥ 1 year of continuous insurance enrollment before index diagnosis and 2 years after. We identified new AM prescriptions using the medication possession ratio. Primary outcomes were corticosteroid use (IBD-only), IBD-related complication (IBD-only), IBD-related surgery (IBD-only), hospitalization, and emergency department (ED) visit(s) within 2 years of diagnosis or starting AM. We calculated adjusted hazard ratios (aHRs) in IBD AM users (for each outcome). We also performed subgroup analyses considering IBD and AM subtype. In the IBD cohort (n = 29,393, 41.4% female; 42.2%CD), 5.2% used AMs. In IBD, AM use was independently associated with corticosteroid use, ED visits, and hospitalizations, but not IBD-related complications. AM use was associated with a decreased risk of surgery. In the control cohort (n = 29,393, 41.4% female), AM use was also independently associated with ED visits and hospitalizations, and there was an increased likelihood of these two outcomes compared to the IBD cohort. In conclusion, while AM use was independently associated with an increased risk of ED visits and hospitalization in IBD, these risks were statistically more common in a matched control cohort. Additionally, AM use was associated with reduced risk of surgery in IBD, demonstrating a potential protective role in this setting.

## Introduction

Anxiety, depression and other psychiatric disorders are frequently diagnosed in the setting of inflammatory bowel disease (IBD)^1,2^, including both Crohn’s disease (CD) and ulcerative colitis (UC). Accordingly, antidepressant medications (AMs) are often prescribed to address mood and anxiety-related disorders in IBD, as well as a variety of associated symptoms frequently described in association with these conditions, including abdominal pain and insomnia^3,4^.

However, recent studies have demonstrated that AMs are associated with serious, adverse effects in certain patient populations, including death^5,6^. Importantly, the most commonly used classes of AMs (e.g., selective serotonin reuptake inhibitors (SSRIs)) are designed to enhance serotonin (5-HT) signaling throughout the body by reducing activity of the selective serotonin reuptake transporter, SERT. This is particularly relevant because IBD has been repeatedly associated with a reduction in the activity and/or availability of SERT and increased levels of 5-HT. Additionally, increased 5-HT availability is associated with inflammation in animal models and human studies of IBD^7–11^.

Thus, there is a concern that AMs, particularly those designed to increase 5-HT availability, may actually aggravate the pathophysiological mechanisms underlying IBD, thereby leading to deleterious consequences for these patient populations. A variety of heterogeneous studies examining IBD patients have, to this point, demonstrated mixed results regarding the overall impact of SSRIs and other 5-HT modulating AMs in these patient populations, depending on the patient population, therapeutic(s) involved and overall study design^3,12,13^. However, no large scale investigation has thus far been designed to specifically evaluate for poor outcomes in this context.

We undertook this study to investigate the relationship between AMs and a variety of key clinical outcomes in the setting of IBD. In particular, we sought to evaluate whether all or particular subtypes of these medications negatively influence the course of these conditions. We also wanted to compare these outcomes to those in other chronic gastrointestinal or auto-inflammatory disorders, as well as the general population. We hypothesized that the use of AMs that specifically target SERT (e.g., SSRIs) would be specifically associated with poor clinical outcomes in IBD.

## Methods

### Data source and cohort selection

We analyzed data from the Merative MarketScan® Commercial Claims and Encounters database. This database consists of reimbursed healthcare claims for over 50 million employees and their dependents annually across all U.S. states and the District of Columbia.

#### Study inclusion criteria

The study populations included all beneficiaries in the database who were 18–63 years of age, had a diagnosis of IBD or were identified as a “control” (as defined below) between January 1, 2006, and December 31, 2012, and who were continuously enrolled in their insurance plan for at least one year prior to the index diagnosis (IBD and “control”) and at least two years after, during the study period. This study was approved by the Penn State College of Medicine Institutional Review Board (PSCOM IRB) under protocol number 00006364. Research conducted under this protocol was performed in accordance with the Declaration of Helsinki and under PSCOM IRB guidelines and regulations. Consent was not required as this study used only de-identified data.

### IBD identification and exclusion of other inflammatory disorders

In addition to the inclusion criteria above, study subjects had (1) at least 2 appropriate ICD-9 diagnoses for IBD [including Crohn’s disease (CD) (ICD-9: 555.0, 555.1, 555.2, or 555.9) and/or ulcerative colitis (UC) (ICD-9: 556.0, 556.1, 556.2, 556.3, 556.4, 556.5, 556.6, 556.8, 556.9)]; and (2) no other auto-inflammatory disease [including rheumatoid arthritis (ICD-9: 714.0), systemic lupus erythematosus (ICD-9: 710.0), psoriasis (ICD-9: 696.1), psoriatic arthritis (696.0), scleroderma (ICD-9: 701.0), systemic sclerosis (ICD-9: 710.1), Sjogren’s disease/syndrome (ICD-9: 710.2), autoimmune hepatitis (ICD-9: 571.42), Hashimoto’s thyroiditis (ICD-9: 245.2), Grave’s disease (ICD-9: 241.0), celiac disease (ICD-9: 579.0), Addison’s disease (ICD-9: 255.41), mixed connective tissue disease (ICD-9: 710.9), polymyositis (ICD-9: 710.4), or polymyalgia rheumatica (ICD-9: 725)].

### Control identification and exclusion of other disorders

Control participants (hereafter referred to as “controls”) were identified using the initial inclusion criteria described above. These individuals also could not have been assigned any ICD-9 codes for IBD (ICD-9: 555.0, 555.1, 555.2, 555.9, 556.0, 556.1, 556.2, 556.3, 556.4, 556.5, 556.6, 556.8, 556.9), RA (ICD-9: 714.0) or one of the chronic auto-inflammatory disorders referred to above.

### Antidepressant use and subtype

In the IBD and control cohorts, we identified new prescriptions of AM (i.e., no AM use in the prior 12-month period) [including selective serotonin reuptake inhibitors (SSRIs) (fluoxetine, fluvoxamine, paroxetine, citalopram, escitalopram, sertraline, vilazadone, vortioxetine), serotonin-norepinephrine reuptake inhibitors (SNRIs) (venlafaxine, desvenlafaxine, duloxetine, levomilnaciprin), serotonin agonist and reuptake inhibitors (SARIs) (nefazadone, trazadone), tricyclics (amitriptyline, desipramine, nortriptyline, protryptaline, amoxapine, doxepine, imipramine, trimipramine), tetracyclics (mirtazapine, maprotiline, asamoxapine), monoamine oxidase inhibitors (selegiline, phenelzine, isocarboxazid, tranylcypromine), bupropion and buspirone] using the medication possession ratio (MPR; 80% adherence over a 6-month period). Of note, no SARI or tetracyclic prescriptions were identified during the search associated with this study so they are not included in this analysis. Additionally, due to the small number of prescriptions for monoamine oxidase inhibitors, bupropion, and buspirone, we decided to aggregate each of these antidepressants into one category described as “other” to be included in the subsequent analysis.

### Study outcomes

In the IBD cohort, the primary study outcome measures were: corticosteroid use (prednisone, methylprednisolone, budesonide, hydrocortisone), IBD-related complication [intestinal stricture (ICD-9: 569.81) and/or fistula (ICD-9: 560.89)], hospitalization, emergency department (ED) visits, and IBD-related surgery (colectomy, subtotal proctocolectomy, total proctocolectomy, proctectomy, enterectomy, ileocecectomy, ileostomy, end ileostomy, loop ileostomy, colostomy, enterostomy) within 2 years of IBD diagnosis or starting AM.

In the control cohort, the primary outcomes of interest were ED visits, hospitalization, and IBD-associated surgery. Note, we excluded individuals who exhibited any of the primary study outcomes during the 12-month period before the index date.

### Ascertainment of potential confounders and statistical analysis

We performed descriptive statistics for the IBD and control study cohorts. Specifically, we summarized the associated demographics, habitation type (rural vs. urban), region in the United States (northeast, west, midwest, unknown), number of clinic visits and, and several co-morbid conditions, including chronic kidney disease (CKD), coronary artery disease (CAD), major depressive disorder (MDD), generalized anxiety disorder (GAD), anxiety state, panic disorder, diabetes mellitus (DM), head injury, hyperlipidemia (HLP), hypertension (HTN), obesity, and stroke (CVA). For the IBD population, we also described IBD-specific medication use [including mesalamine or equivalents (mesalamine, sulfasalazine, balsalazide, olsalazine), immunomodulators (azathioprine, 6-mercaptopurine (6-MP), methotrexate), and/or biologics (infliximab, adalimumab, certolizumab, golimumab, ustekinumab, natalizumab, vedolizumab)].

Cox proportional hazards regression analysis was then used to calculate adjusted hazard ratios (aHRs) and corresponding 95% confidence intervals (CIs) to determine the risk of developing one of the outcomes described above in two patient sub-cohorts (IBD AM Users and IBD non-AM Users). In the multivariable model, we adjusted for the following potential confounders: age, sex, habitation, number of clinic visits, IBD medication use and comorbidity as described above [the latter evaluated on a binary (i.e., yes/no) basis]. We also performed subgroup analyses considering the type of IBD (e.g., CD, UC) and AM [e.g., SSRI, SNRI, tricyclics, and “other” (as defined above)]. We then performed separate multivariable analysis for the control cohort (again, comparing AM Users and non-AM Users), accounting for age, sex, habitation, number of clinic visits and comorbidity (using the same diagnoses described above). Effect modification was tested by including the multiplicative interaction term between antidepressant use (“yes” versus “no”) and IBD status (“yes” versus “no”) and testing the -2log-likelihood ratio (-2 LL) differences between the full and reduced models. To further test the robustness of our results, we performed a sensitivity analysis in the whole IBD cohort to minimize potential confounding bias. We performed this analysis by determining the inverse probability of anti-depressant use weighting using the propensity score, which was calculated using the aforementioned covariates in the final model, to balance baseline data between individuals with and without anti-depressant use. SAS statistical software (version 9.4, SAS Institute, Cary, NC) was used to perform all statistical analysis using 2-tailed *P* < 0.05 as the significance level.

## Results

### Demographic characteristics and antidepressant use in IBD

We identified 29,393 people with an established diagnosis of IBD (41.4% female; 42.2% CD) (Table 1). In this cohort, 5.2% used at least one AM during the follow-up period (SSRI = 4.6%, SNRI = 1.1%, Other = 1.0%, Tricyclics = 0.4%). The unmatched IBD AM user cohort trended toward being younger (*p* = 0.08), and were significantly more likely to be female and to have certain comorbidities, including GAD, MDD, and prior head injury (Table 1), when compared to IBD non-users. IBD patients who used AMs were also more likely to use corticosteroids and each major subtype of IBD-targeted medications. Finally, AM users exhibited a higher mean number of clinic visits (Table 1).

**Table 1:** Demographic and clinical characteristics of antidepressant medication users and non-users among IBD patients.

Parameter	No AM use (n = 27,865)	AM use (n = 1,528)	*P* value
Age [years (mean ± SD)]	44.7 ± 11.1	44.2 ± 11.2	0.08
Gender (%)			< 0.0001
Male	16,542 (59.4)	682 (44.6)	
Female	11,323 (40.6)	846 (55.4)	
Residential Setting (%)			0.003
Rural	3,819 (13.7)	251 (16.4)	
Urban	24,046 (86.3)	1,277 (83.6)	
US Regions			< 0.0001
South	10,581 (38.0)	574 (37.6)	
West	4123 (14.8)	236 (15.5)	
Midwest	7206 (25.9)	488 (31.9)	
Northeast	5650 (20.3)	211 (13.8)	
Unknown	305 (1.1)	19 (1.2)	
Diabetes mellitus (%)	1557 (5.6)	74 (4.8)	0.22
Hypertension (%)	4460 (16.0)	230 (15.1)	0.32
Obesity (%)	363 (1.3)	24 (1.6)	0.37
Hyperlipemia (%)	5650 (20.3)	299 (19.6)	0.50
Generalized Anxiety Disorder (%)	611 (2.2)	98 (6.4)	< 0.0001
Major Depressive Disorder (%)	726 (2.6)	130 (8.5)	< 0.0001
Stroke (%)	180 (0.7)	13 (0.9)	0.33
Coronary Artery Disease (%)	599 (2.2)	34 (2.2)	0.84
Head Injury (%)	30 (0.1)	5 (0.3)	0.02
Chronic Kidney Disease (%)	140 (0.5)	9 (0.6)	0.64
Corticosteroid Use (%)	11,118 (39.9)	994 (65.1)	< 0.0001
Clinic Visits (mean ± SD)	7.3 ± 8.9	9.3 ± 10.7	< 0.0001
IBD related medications‡			
Mesalamine use (%)	14,945 (53.6)	1071 (70.1)	< 0.0001
Immunomodulator use (%)	4747 (17.0)	383 (25.1)	< 0.0001
Biologic use (%)	1488 (5.3)	162 (10.6)	< 0.0001

AM, antidepressant medication; SD, standard deviation.

^‡^IBD patients only.

In the CD cohort (n = 12,417), 5.8% used at least one AM during the follow-up period. CD AM users were more likely to be female, and to have several comorbidities, including GAD, MDD and head injury (Table 2). CD AM users were more likely to use corticosteroids and each major subtype of IBD-targeted medication. They also exhibited a higher mean number of clinic visits (Table 2).

**Table 2:** Demographic and clinical characteristics of antidepressant medication users and non-users among CD patients.

Parameter	No AM use (n = 11,697)	AM use (n = 720)	*P* value
Age [years (mean ± SD)]	43.9 ± 11.5	43.6 ± 11.6	0.56
Gender (%)			< 0.0001
Male	6740 (57.6)	305 (42.4)	
Female	4957 (42.4)	415 (57.6)	
Residential setting (%)			0.02
Rural	1642 (14.0)	124 (17.2)	
Urban	10,055 (86.0)	596 (82.8)	
US Regions			0.0002
South	4492 (38.4)	273 (37.9)	
West	1424 (12.2)	96 (13.3)	
Midwest	3193 (27.3)	238 (33.1)	
Northeast	2445 (20.9)	105 (14.6)	
Unknown	143 (1.2)	8 (1.1)	
Diabetes Mellitus (%)	542 (4.6)	37 (5.1)	0.53
Hypertension (%)	1823 (15.6)	103 (14.3)	0.36
Obesity (%)	151 (1.3)	10 (1.4)	0.82
Hyperlipemia (%)	2104 (18.0)	123 (17.1)	0.54
Generalized Anxiety Disorder (%)	257 (2.2)	45 (6.3)	< 0.0001
Major Depressive Disorder (%)	326 (2.8)	64 (8.9)	< 0.0001
Stroke (%)	79 (0.7)	6 (0.8)	0.62
Coronary Artery Disease (%)	224 (1.9)	15 (2.1)	0.75
Head Injury (%)	13 (0.1)	3 (0.4)	0.03
Chronic Kidney Disease (%)	80 (0.7)	7 (1.0)	0.37
Corticosteroid Use (%)	4586 (39.2)	453 (62.9)	< 0.0001
Clinic Visits (mean ± SD)	7.8 ± 9.1	9.7 ± 11.1	< 0.0001
IBD related medications‡			
Mesalamine use (%)	5164 (44.2)	443 (61.5)	< 0.0001
Immunomodulator use (%)	2749 (23.5)	228 (31.7)	< 0.0001
Biologic use (%)	1083 (9.3)	129 (17.9)	< 0.0001

AM, antidepressant medication; SD, standard deviation.

^‡^IBD patients only.

In the UC cohort (n = 17,784), 4.5% used at least one AM during the follow-up period. UC AM users were also more likely to be female and to have exhibited GAD, MDD, and trended toward being less likely to have diabetes mellitus (DM) (*p* = 0.05) (Table 3). As with the CD cohort, UC AM users were more likely to use corticosteroids as well as each major subtype of IBD-targeted medication. They also demonstrated a significantly higher mean number of clinic visits (Table 3).

**Table 3:** Demographic and clinical characteristics of antidepressant medication users and non-users among UC patients.

Parameter	No AM use (n = 16,168)	AM use (n = 808)	*P* value
Age [years (mean ± SD)]	45.2 ± 10.8	44.6 ± 10.9	0.12
Gender (%)			< 0.0001
Male	9802 (60.6)	377 (46.7)	
Female	6366 (39.4)	431 (53.3)	
Residential setting (%)			0.07
Rural	2177 (13.5)	127 (15.7)	
Urban	13,991 (86.5)	681 (84.3)	
US Regions			< 0.0001
South	6089 (37.7)	301 (37.3)	
West	2699 (16.7)	140 (17.3)	
Midwest	4013 (24.8)	250 (30.9)	
Northeast	3205 (19.8)	106 (13.1)	
Unknown	162 (1.0)	11 (1.4)	
Diabetes mellitus (%)	1015 (6.3)	37 (4.6)	0.05
Hypertension (%)	2637 (16.1)	127 (15.7)	0.66
Obesity (%)	212 (1.3)	14 (1.7)	0.31
Hyperlipemia (%)	3546 (21.9)	176 (21.8)	0.92
Generalized anxiety disorder (%)	354 (2.2)	53 (6.6)	< 0.0001
Major depressive disorder (%)	400 (2.5)	66 (8.2)	< 0.0001
Stroke (%)	101 (0.6)	7 (0.9)	0.40
Coronary artery disease (%)	375 (2.3)	19 (2.4)	0.95
Head Injury (%)	17 (0.1)	2 (0.3)	0.24
Chronic kidney disease (%)	60 (0.4)	2 (0.3)	0.57
Corticosteroid use (%)	6532 (40.4)	541 (67.0)	< 0.0001
Clinic visits (mean ± SD)	7.0 ± 8.7	9.0 ± 10.3	< 0.0001
IBD related medications‡			
Mesalamine use (%)	9781 (60.5)	628 (77.7)	< 0.0001
Immunomodulator use (%)	1998 (12.4)	155 (19.2)	< 0.0001
Biologic use (%)	405 (2.5)	33 (4.1)	0.006

AM, antidepressant medication; SD, standard deviation.

^‡^IBD patients only.

### Multivariable analysis of AM use in whole IBD and IBD sub-cohorts

After adjusting for age, sex, number of clinic visits, IBD medication use, and major medical comorbidities (including GAD and MDD), we found that AM use was independently associated with corticosteroid use, hospitalizations, and ED visits, in the whole IBD cohort (Table 4). There was no significant association between AM use and IBD-associated complications or IBD-associated surgery. In order to assess the robustness of these findings, we also performed a sensitivity analysis of the results using propensity scoring (incorporating the same variables as those described above). This demonstrated, as above, that AM use was independently associated with corticosteroid use, hospitalizations, and ED visits but not IBD-associated complications. Of note, AM use was inversely associated with IBD-associated surgery (Table 5).

**Table 4:** Cox proportional hazards ratios (HR) evaluating clinical outcomes associated with antidepressant use in IBD patients.

Clinical outcomes	No AM use	AM use	*P* value
Corticosteroid use	1(ref.)	2.23 (2.06, 2.42)	< 0.0001
IBD-Associated complication	1(ref.)	1.22 (0.79, 1.88)	0.37
Hospitalization	1(ref.)	1.15 (1.01, 1.32)	0.04
Emergency department visit	1(ref.)	1.27 (1.15, 1.40)	< 0.0001
IBD-Associated surgery	1(ref.)	0.78 (0.49, 1.24)	0.30

Each model was adjusted for age, gender, residence type, US region, office visits, IBD status (yes vs. no), co-morbidity (anxiety, depression, diabetes, hypertension, obesity, hyperlipidemia, stroke, coronary artery disease, head injury, chronic kidney disease, each yes vs. no), and corticosteroid use (the latter except when the impact of corticosteroid use was itself being evaluated).

**Table 5:** Hazards ratios (HR) derived from a sensitivity analysis, weighting by inverse propensity score, evaluating clinical outcomes associated with antidepressant use in IBD patients.

Clinical outcomes	No AM use	AM use	*P* value
Corticosteroid use	1(ref.)	2.16 (2.09, 2.22)	< 0.0001
IBD-associated complication	1(ref.)	1.12 (0.97, 1.30)	0.14
Hospitalization	1(ref.)	1.08 (1.03, 1.13)	0.002
Emergency department visits	1(ref.)	1.23 (1.19, 1.27)	< 0.0001
Surgery	1(ref.)	0.73 (0.63, 0.84)	< 0.0001

Each model was adjusted for age, gender, residence type, US region, office visits, IBD status (yes vs. no), co-morbidity (anxiety, depression, diabetes, hypertension, obesity, hyperlipidemia, stroke, coronary artery disease, head injury, chronic kidney disease, each yes vs. no), and corticosteroid use (the latter except when the impact of corticosteroid use was itself being evaluated).

We also performed a multivariable regression analysis in the CD sub-cohort, and found that AM use was independently associated with corticosteroid use, hospitalizations, and ED visits (see Supplemental Table S1). There was also no significant association between AM use in the CD sub-cohort with IBD complications or IBD-associated surgery. When this analysis was performed in the UC sub-cohort, AM use was also independently associated with corticosteroid use and ED visits (see Supplemental Table S2). There were no significant associations between AM use in the UC sub-cohort and hospitalizations, IBD-associated surgery, or IBD-associated complications.

We also evaluated the impact of different AM subtypes on clinical outcomes in the whole IBD cohort. SSRIs, SNRIs, and Tricyclics were each independently associated with corticosteroid use and ED visits, while other AMs were associated with only corticosteroid use (Table 6). No AM subtype was associated with hospitalizations, IBD-associated complications or IBD-associated surgery (Table 6).

**Table 6:** Cox proportional hazards ratios (HR) evaluating clinical outcomes associated with antidepressant subtypes among IBD patients.

Clinical outcomes	No AM Use	SNRI	SSRI	Tricyclics	Others
Corticosteroid use	1(ref.)	2.38 (1.98, 2.86)	2.16 (1.96, 2.38)	2.41 (1.67, 3.47)	2.53 (2.08, 3.07)
Complication	1(ref.)	0.93 (0.30, 2.92)	1.17 (0.70, 1.98)	1.22 (0.17, 8.72)	1.47 (0.55, 3.97)
Hospitalization	1(ref.)	1.29 (0.96, 1.73)	1.17 (0.99, 1.37)	1.30 (0.72, 2.36)	1.04 (0.73, 1.48)
Emergency room visit	1(ref.)	1.64 (1.34, 2.01)	1.23 (1.09, 1.39)	1.63 (1.08, 2.45)	1.13 (0.87, 1.47)
Surgery	1(ref.)	0.72 (0.23, 2.26)	0.67 (0.37, 1.22)	0.93 (0.13, 6.62)	1.16 (0.43, 3.12)

Each model was adjusted for age, gender, residence type, US region, office visits, IBD status (yes vs. no), co-morbidity (anxiety, depression, diabetes, hypertension, obesity, hyperlipidemia, stroke, coronary artery disease, head injury, chronic kidney disease, each yes vs. no), and corticosteroid use (the latter except when the impact of corticosteroid use was itself being evaluated).

### Demographic characteristics and antidepressant use in a control cohort

Using the criteria described above, we identified 29,393 individuals who qualified as controls (441.4% female) (Table 7). In this cohort, 4.5% used at least one AM during the follow-up period (significantly less than the IBD cohort, *p* < 0.0001) (SSRI = 3.8%, SNRI = 0.7%, Other = 0.8%, Tricyclics = 0.2%). Of note, AM use was less frequent in controls when compared to the IBD cohort (4.5% vs. 5.2%, *p* < 0.0002). Controls using AMs were more likely to exhibit hypertension, GAD, MDD and prior head injury, and trended toward being more likely to have DM (*p* = 0.08). They also exhibited a higher mean number of clinic visits (Table 7).

**Table 7:** Demographic and clinical characteristics of antidepressant medication users and non-users among control patients.

Parameter	No AM use (n = 28,057)	AM use (n = 1,336)	*P* value
Age [years (mean ± SD)]	44.7 ± 11.1	44.5 ± 10.5	0.55
Gender (%)			< 0.0001
Male	16,682 (59.5)	542 (40.6)	
Female	11,375 (40.5)	794 (59.4)	
Residential Setting (%)			0.002
Rural	7,533 (26.9)	411 (30.8)	
Urban	20,524 (73.2)	925 (69.2)	
US Regions			0.008
South	17,281 (61.6)	848 (63.5)	
West	1,416 (5.1)	61 (4.6)	
Midwest	6,830 (24.3)	340 (25.5)	
Northeast	2,502 (8.9)	84 (6.3)	
Unknown	28 (0.1)	3 (0.2)	
Diabetes Mellitus (%)	1,750 (6.2)	99 (7.4)	0.08
Hypertension (%)	4,746 (16.9)	265 (19.8)	0.006
Obesity (%)	329 (1.2)	15 (1.1)	0.87
Hyperlipemia (%)	4,943 (17.6)	247 (18.5)	0.42
Generalized Anxiety Disorder (%)	207 (0.7)	45 (3.4)	< 0.0001
Major Depressive Disorder (%)	208 (0.7)	46 (3.4)	< 0.0001
Stroke (%)	127 (0.5)	7 (0.5)	0.71
Coronary Artery Disease (%)	537 (1.9)	23 (1.7)	0.62
Head Injury (%)	17 (0.1)	4 (0.3)	0.01
Chronic Kidney Disease (%)	89 (0.3)	5 (0.4)	0.62
Corticosteroid Use (%)	12,016 (42.8)	790 (59.1)	< 0.0001
Clinic Visits (mean ± SD)	4.4 ± 7.3	6.0 ± 7.5	< 0.0001

AM, antidepressant medication; SD, standard deviation.

### Multivariable analyses comparing matched IBD and control cohorts

On multivariable analysis, we found that control AM users exhibited a higher risk of ED visits and hospitalization, but not IBD-associated surgeries (Table 8). We also compared clinical outcomes between the IBD and control cohorts. After adjusting for age, sex, number of clinic visits, and major medical comorbidities (including GAD and MDD), we found that control AM users also exhibited a higher risk of ED visits and hospitalization (Table 8).

**Table 8:** HRs of clinical outcomes according to anti-depressant use status, comparing IBD and control cohorts.

	No AM use	AM use	*P*-interaction
ED Visit			
IBD	1(ref.)	1.26 (1.14, 1.39)	0.005
Controls	1(ref.)	1.57 (1.40, 1.76)	
Surgery			
IBD	1(ref.)	0.79 (0.50, 1.26)	0.96
Controls	1(ref.)	0.74 (0.10, 5.45)	
Hospitalization			
IBD	1(ref.)	1.16 (1.02, 1.33)	0.01
Controls	1(ref.)	1.54 (1.29, 1.84)	

Each model was adjusted for age, gender, residence type, US region, office visits, co-morbidity (anxiety, depression, diabetes, hypertension, obesity, hyperlipidemia, stroke, coronary artery disease, head injury, chronic kidney disease, each yes vs. no), and corticosteroid use (the latter except when the impact of corticosteroid use was itself being evaluated).

## Discussion

In this study, both IBD and non-IBD control patients taking antidepressants exhibited increased likelihood of several poor clinical outcomes. In IBD, this included an increased probability of corticosteroid use, ED visits, and hospitalizations, though not IBD complications (e.g., intra-abdominal strictures and/or fistulae). Interestingly, in the whole IBD cohort, there was an inverse association between AM use and IBD-associated surgery. Importantly, when comparing the IBD and control cohorts, control patients actually had a significantly higher likelihood of visiting the ED and being hospitalized. When considering AM subtype, each was significantly associated with increased likelihood of corticosteroid use, while most were also associated with ED visits in IBD. No AM subtype was associated with IBD-related complications or surgery. When considering IBD subtype, CD and UC AM users were more likely to use corticosteroids and to visit the ED. CD AM users were also more likely to be hospitalized, while UC AM users were not. Neither CD nor UC AM users demonstrated an increased likelihood of IBD-associated complications or surgery.

These findings are novel and worthy of further consideration for several reasons. While adverse outcomes related to AM use, including death, have been reported in other groups^5,6^, this is the first large-scale, population-based investigation to demonstrate that AM use is associated with poor outcomes in any chronic gastrointestinal and/or inflammatory condition. It is also one of the first studies to compare IBD patients and a non-IBD control population in this context. Importantly, this study also demonstrated that the relative risk of key poor outcomes in IBD is less than that of healthy controls (ED visits and hospitalization). Importantly, it was also demonstrated that IBD-related complications (intestinal stricture and/or fistula) was not more likely, and IBD-associated surgery was actually less likely in the setting of AM use. There was also no significant differentiation in these outcomes when considering AM subtype. These findings suggest AMs, including serotonin-targeting agents, do not necessarily pose an increased risk to IBD patients relative to society at large. In fact, considering the demonstration that IBD-associated surgery was less likely in IBD AM users, it is possible there may be a protective effect related to antidepressants in this setting. This is an important revelation, particularly considering the widespread use of these medications in the setting of IBD and the general population, especially in patients with comorbid mood and anxiety disorders, abdominal pain and/or other symptoms (which are all commonly found in the setting of IBD)^14^.

There are several limitations to this study. It was a large but retrospective study and subject to all of the potential biases associated with that design. While we could identify patient deaths, we could not determine their cause. This was also true for the underlying drivers of ED visits and hospitalization. Additionally, although we considered multiple potential comorbidities, we may have missed other contributory conditions. Some of the total numbers for specific outcomes (e.g., hospital death), and certain health conditions (e.g., obesity) were also relatively small and this made comparisons among subgroups challenging, particularly when performing AM subtype analyses. It was, thus, impossible to establish cause-and-effect relationships or to definitively determine whether one AM subtype was more problematic. Separately, data analyzed in this study was derived from 2006 to 2012, a period of time when use of biologic and other more potent IBD-directed therapies, was less common. This could have influenced relative disease control and overall rates of anxiety and depression in the IBD cohorts. Thus, it would be reasonable to analyze data derived from a more recent time period to determine whether the rates of psychiatric illness and AM use had changed. Finally, we did not have large enough sub-cohorts to effectively examine the impact of certain AM types (i.e., monoamine oxidase inhibitors, SARIs, tetracyclics, bupropion, buspirone). Certainly, it will be important to re-examine each of these factors in other cohorts, under more carefully controlled circumstances, to clarify whether there is a link between them and AM use.

The results of this study do not allow for a definitive recommendation to endorse or oppose the use of AMs in the setting of IBD. Our findings do raise important questions about the overall safety of these medications. However, the findings of this study suggest that they are not more dangerous in IBD, and they may actually be somewhat protective (e.g., against surgery). It is also important to note that antidepressants have been used to treat a wide variety of conditions for decades, and there is significant evidence for the positive impact they can make in IBD. Previous studies have demonstrated this relatively positive impact not only in relation to comorbid psychiatric conditions but also (in at least selected sub-populations) on disease course and/or gastrointestinal symptom experience in IBD^3,12,15^. Irrespective of these findings, and considering the relatively high co-incidence of anxiety and depression exhibited in these disorders^1,2^, it would also be very challenging to completely avoid AMs in these populations anyway.

The findings reported here are worthy of careful consideration. As noted above, certain negative outcomes were more likely in AM users in general. These results were relatively consistent, and exhibited in both the IBD (including both major subtypes) and control populations. However, the risks appeared to be lower in IBD, and there was also evidence that AM use might be protective against surgery in this setting. Further large-scale studies should be undertaken in both IBD and otherwise healthy individuals to more carefully evaluate the associations demonstrated in this study. If substantiated, healthcare providers utilizing AMs may need to reconsider the overall safety and utility of these agents in their practice, at least until more clarity is gained regarding the nature of these risks. Until then, IBD providers may take solace in the finding that antidepressants do not appear to be any more problematic for the people they care for when compared to other individuals, and may even be beneficial in this patient population beyond mental health.

### Supplementary Information

Supplementary Tables.

## Data Availability

All data generated during this study are included in this manuscript. There are no additional data associated with this article.
