# Supplementary material for: Clinical outcomes associated with antidepressant use in inflammatory bowel disease patients and a matched control cohort
Source: Sci Rep. 2024 Jan 11;14:1060. doi: 10.1038/s41598-024-51282-6 (PMC10784571; doi:10.1038/s41598-024-51282-6)
Supplement: Supplementary file 1 — Supplementary Tables. [file 41598_2024_51282_MOESM1_ESM.pdf]

Clinical Outcomes Associated with Antidepressant Use in Inflammatory Bowel Disease Patients and a Matched Control Cohort

Djibril M. Ba[1,2], Sanjay Yadav[3], Guodong Liu[1,2], Douglas L. Leslie[1,2], Kent E. Vrana[4], Matthew D. Coates[4,5*]

[1]Department of Public Health Sciences, Pennsylvania State University College of Medicine, Hershey, PA, USA

[2]Center for Applied Studies in Health Economics (CASHE), Pennsylvania State University College of Medicine, Hershey, PA, USA

[3]Department of Psychiatry, Pennsylvania State University College of Medicine, Hershey, PA, USA

[4]Department of Pharmacology, Pennsylvania State University College of Medicine, Hershey, PA, USA

[5]Department of Medicine, Division of Gastroenterology & Hepatology, Pennsylvania State University College of Medicine, Hershey, PA, USA

Supplemental Table S1. Cox Proportional Hazards Ratios (HR) Evaluating Clinical Outcomes Associated with Antidepressant Use in the Matched CD Cohort.

Clinical Outcomes	No AM Use	AM Use	P value
**Corticosteroid Use**	1(ref.)	2.19 (1.94, 2.47)	<0.0001
**Complications**	1(ref.)	1.01 (0.61, 1.66)	0.98
**Hospitalization**	1(ref.)	1.24 (1.14, 1.36)	<0.0001
**Emergency Room Visit**	1(ref.)	1.32 (1.15, 1.51)	<0.0001
**Surgery**	1(ref.)	0.87 (0.52, 1.45)	0.59

Note: Each model was adjusted for age, gender, residence type, US region, office visits, IBD status (yes vs. no), co-morbidity (anxiety, depression, diabetes, hypertension, obesity, hyperlipidemia, stroke, coronary artery disease, head injury, chronic kidney disease, each yes vs. no), and corticosteroid use (the latter except when the impact of corticosteroid use was itself being evaluated).

Supplemental Table S2. Cox Proportional Hazards Ratios (HR) Evaluating Clinical Outcomes Associated with Antidepressant Use Among UC patients.

Clinical Outcomes	No AM Use	AM Use	P value
**Corticosteroid Use**	1(ref.)	2.27 (2.04, 2.54)	<0.0001
**Complication**	1(ref.)	1.91 (0.80, 4.55)	0.14
**Hospitalization**	1(ref.)	1.10 (0.91, 1.35)	0.33
**Emergency Room Visit**	1(ref.)	1.20 (1.04, 1.38)	0.01
**Surgery**	1(ref.)	0.44 (0.14, 1.40)	0.17

Note: Each model was adjusted for age, gender, residence type, US region, office visits, IBD status (yes vs. no), co-morbidity (anxiety, depression, diabetes, hypertension, obesity, hyperlipidemia, stroke, coronary artery disease, head injury, chronic kidney disease, each yes vs. no), and corticosteroid use (the latter except when the impact of corticosteroid use was itself being evaluated).
